# Project SoL—A Community-Based, Multi-Component Health Promotion Intervention to Improve Eating Habits and Physical Activity among Danish Families with Young Children. Part 1: Intervention Development and Implementation

**DOI:** 10.3390/ijerph15061097

**Published:** 2018-05-28

**Authors:** Ulla Toft, Paul Bloch, Helene C. Reinbach, Lise L. Winkler, Tine Buch-Andersen, Jens Aagaard-Hansen, Bent Egberg Mikkelsen, Bjarne Bruun Jensen, Charlotte Glümer

**Affiliations:** 1 Center for Clinical Research and Prevention, Capital Region, Nordre Fasanvej 57, 2000 Frederiksberg, Denmark; lawaetzlise@gmail.com (L.L.W.); tine.buch-andersen@regionh.dk (T.B.-A.); charlotte.glumer@suf.kk.dk (C.G.); 2 Steno Diabetes Center Copenhagen, Health Promotion, Niels Steensens Vej 2-4, 2820 Gentofte, Denmark; paul.bloch@regionh.dk (P.B.); jens.aagaard-hansen@regionh.dk (J.A.-H.); bjarne.bruun.jensen@regionh.dk (B.B.J.); 3 Department of Development and Planning, Aalborg University, A.C. Meyers Vænge 15 2nd floor, 2450 Copenhagen SV, Denmark; hcr@plan.aau.dk; 4 MRC Developmental Pathways for Health Research Unit, Faculty of Health Sciences, University of the Witwatersrand, 1 Jan Smuts Avenue, Braamfontein 2000, Johannesburg, South Africa; 5 Department of Learning & Philosophy, Aalborg University, A.C. Meyers Vænge 15, 3rd floor, 2450 Copenhagen SV, Denmark; bemi@learning.aau.dk

**Keywords:** action research, children, community, complex interventions, Denmark, families, health promotion, mass media, social media, mixed methods, realistic evaluation, schools, supermarkets

## Abstract

Project SoL was implemented over a period of four years from 2012–2015 with the aim to promote healthy eating and physical activity among families with children aged 3–8 years, living in selected communities in two Danish municipalities. This was done by applying the supersetting approach to implement complex multi-component interventions in a participatory, coordinated, and integrated manner in childcare centres, schools, and supermarkets in three local communities, as well as in local media during a 19-month period in the Regional Municipality of Bornholm, which served as the intervention site. The matching municipality of Odsherred served as a control site based on its similarity to Bornholm regarding several socio-demographic and health indicators. The present paper describes the design of Project SoL as well as the processes of developing and implementing its complex interventions. Moreover, the theoretical and conceptual framework of the project is described together with its organisational structure, concrete activities, and sustainability measures. The paper discusses some of the key lessons learned related to participatory development and the implementation of a multi-component intervention. The paper concludes that coordinated and integrated health promotion activities that are implemented together with multiple stakeholders and across multiple settings in the local community are much more powerful than individual activities carried out in single settings. The supersetting approach was a useful conceptual framework for developing and implementing a complex multi-component health promotion intervention and for fostering ownership and sustainability of the intervention in the local community. The research and evaluation approach of the project is described in a separate paper (Part 2).

## 1. Introduction

### 1.1. Non-Communicable Diseases and Health Promotion

Sedentarism, inadequate physical activity, and unhealthy dietary habits are some of the main risk factors for the increasing prevalence of obesity, cardiovascular diseases, and type 2 diabetes [1]. In particular, unhealthy lifestyle among children and adolescents is of great concern [2,3]. The potential for prevention is greatest among children because many lifestyle practices, such as dietary habits and physical activity, are founded in early childhood and may be tracked into adulthood [4,5,6,7]. 

Individual-level approaches to promote healthier behaviour have only shown transient impact on health indicators and, furthermore, increase social inequality in health [8,9]. Therefore, in accordance with the recommendations of the WHO Ottawa Charter [10], rather than focusing narrowly on the health behaviour of the individual, the work on health promotion and disease prevention should be based on the understanding that people’s health and well-being is strongly influenced by the social, cultural, and environmental contexts of people’s everyday life [11,12]. Consequently, there has been an increasing focus on the promotion of health through healthy public policies, supportive environments, and community actions. The Ottawa Charter states that “health is created and lived by people within the settings of their everyday life; where they learn, work, play and love” [10]. Hence, significant effort has gone into interventions in key community institutions, such as schools [13,14,15], pre-schools [16,17], worksites [18,19], and food stores [20,21], and studies using “the settings approach” [10] have demonstrated the effects of interventions on food purchase and consumption [22,23], as well as on physical activity [24]. However, interventions implemented within a single setting tend to rely on intensive short-term activities, and may have limited sustainability [25]. Therefore, there is increasing interest in coordinated and integrated interventions in multiple settings in the local community [26,27,28,29,30].

### 1.2. Project Sol

Project SoL—from the Danish ‘Sundhed og Lokalsamfund’ (Health and Local Community) is one example of a health promotion project, which was carried out in multiple settings in the local community. The project was based on the supersetting approach [31] and targeted schools, childcare centres, and supermarkets as well as the local mass media and social media. In accordance with the supersetting approach, the intervention was implemented in a coordinated and integrated manner in several everyday life settings to promote intensity, impact, synergy, and sustainability.

The present paper describes the development and design of the intervention of Project SoL and its implementation. The research and evaluation approach of the project is described in a separate paper (hereinafter referred to as “Part 2: Research and Evaluation”). Furthermore, outcomes and results of Project SoL are described in separate papers.

## 2. Intervention Methods

### 2.1. Overall Intervention Aim and Design

Project SoL was a research and development project that aimed to promote healthier lifestyles among Danish children aged 3–8 years and their families. Focus was on promoting healthier dietary habits and physical activity, as well as on mobilizing local community resources, strengthening social networks, and reducing social inequality. The project was carried out in Denmark over a four-year period from 2012 to 2015. The first part of the project included a 19-months intervention period during which a multi-component intervention was implemented in multiple settings, including childcare centres, schools, supermarkets, and local media, in three local communities in the Regional Municipality of Bornholm. The project was designed as a quasi-experimental study with matched intervention and control communities. Three equivalent communities in Odsherred Municipality functioned as non-intervention control communities for the quantitative evaluation of the project. After the end of the intervention period in Bornholm, the applied intervention approach was implemented over a period of one year in Odsherred Municipality. This was done to test whether the multicomponent intervention approach was transferable to other community contexts. This last part of the project is not described further in the present paper.

The Regional Municipality of Bornholm is an island with a land mass of 588 square kilometres and a population of approximately 42,000 inhabitants. Odsherred Municipality (comparison site), is an area of 355 square kilometres with about 32,500 inhabitants. Bornholm and Odsherred were purposely selected as the first level of sampling due to their similar socio-demographic characteristics, including high proportions of citizens with a low socioeconomic position and high prevalence of health risk factors and non-communicable diseases [32,33]. Table 1 gives an overview of important characteristics of the two municipalities.

The second level of sampling was the community level. Three local communities in Bornholm were selected in collaboration with the officials in the municipality and based on a need to include a school, a childcare centre, and supermarkets in the same local community. A local community was defined geographically as a town and its catchment area. The selected local communities on Bornholm were: Nexø, Hasle, and Allinge-Sandvig. A similar approach was used to select three local communities in Odsherred: Asnæs, Højby, and Egebjerg.

The main target group was families with children enrolled in the participating childcare centres (age range 3–6 years) and primary schools, grade zero to two (age range 6–8 years). Approximately 440 and 420 children were eligible to participate in the three local communities on Bornholm and in Odsherred, respectively.

Several local stakeholders were involved in the development and implementation of activities including professionals within the municipality, primary schools, after-school centres, childcare centres, supermarkets, media, and a number of civil society organizations and resource persons with expertise in nutrition, cooking, recreation, and physical activity.

### 2.2. Theoretical and Conceptual Framework

With inspiration from the setting approach [34], Project SoL developed, applied, and tested a new participatory intervention strategy, the supersetting approach [31], to mobilize local communities for public health action. This involves the coordinated engagement of multiple stakeholders in multiple community settings targeting a common overall goal, such as improved health in a population group. The supersetting approach includes five principles, namely (1) integration to ensure that activities are implemented across the boundaries of specific settings; (2) participation to ensure that people are motivated to take ownership of processes of developing and implementing activities; (3) empowerment or action competence to ensure that people acquire skills and competences to express and act on their visions and aspirations; (4) context to ensure that everyday life challenges of citizens and professionals are respected and considered in planning activities; and (5) knowledge to ensure that scientific knowledge is used to inform action and that scientific knowledge is produced from action. The supersetting approach builds on the use of resources embedded in local community settings and on the strengths of social engagement and local ownership as drivers of change processes. The supersetting approach is based on an ecological model and recognizes that children and their families are deeply embedded in social, environmental, and cultural contexts [35]. Therefore, health outcomes and behaviours are results of complex interactions between the knowledge, motivations, and attitudes of the children and their families, and the social and physical surroundings of the local community in which they live. This calls for a holistic perspective to change potentials and developmental processes with a starting point in the circumstances of people’s everyday life. It also calls for multi-component interventions addressing multiple settings and levels in a whole-systems perspective.

A main principle of Project SoL’s intervention was to combine different health promotion and prevention strategies including mass strategies working through information and education, environmental strategies working through structural changes in the environment, person-oriented strategies targeting individuals and social strategies working through social mobilization, interaction, and networking. The hypothesis was that the application of a combination of intervention strategies would generate synergistic effects that cannot be achieved by means of single-stranded interventions [36,37].

### 2.3. Organizational Structure

**Figure 1:**
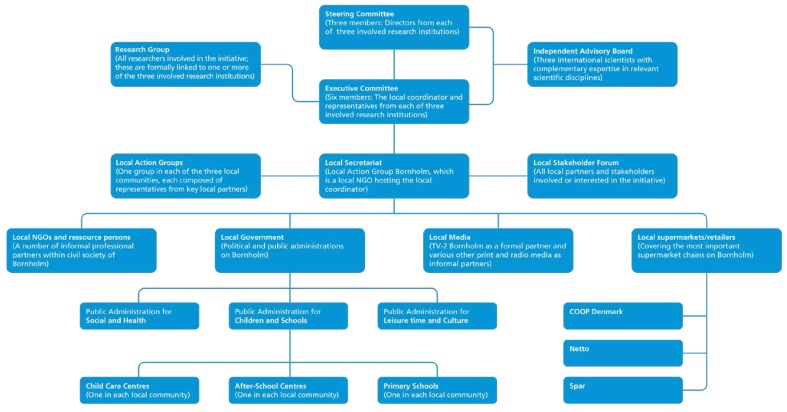
The organizational structure of Project SoL.

The organizational structure of Project SoL is shown in Figure 1. The various boxes represent partners in addition to settings, coordination groups, and an independent advisory committee. Thus, the project was organized as a formalized partnership with the three participating research institutions (Aalborg University (AAU), Steno Diabetes Center (SDC), Research Centre for Prevention and Health (RCPH), and key local stakeholders, including three departments within local government (health/social services, education/day-care and leisure/prevention), a local NGO (Lokal Aktionsgruppe Bornholm; hosting the local coordinator of the initiative) engaged in community development, three supermarket chains with outlets/shops in the involved communities, and the local TV station. Furthermore, during the project period three Local Action Groups were established for professionals (e.g., school teachers, shop owners, fitness instructors etc.) and citizens working and/or living in the involved communities (described further below). 

An executive committee consisting of the local coordinator in addition to a senior researcher from each research institution functioned as the driver of the project with responsibility for the day-to-day coordination and planning of both the intervention and the research processes. The local coordinator headed the local secretariat. The executive committee and the local secretariat were in close dialogue and interaction regarding the development and implementation of the project during the project period. This also included co-creative processes of forming the intervention with various local stakeholders within the municipality, the civil society, businesses, and the local media, in addition to numerous informal meetings, phone calls and e-mails. Hence, it was through the executive committee that development and research agendas were synchronized and more widely communicated within the organizational structure. The Steering Committee consisted of directors from each of the three involved research institutions. The Steering Committee gave overall strategic guidance to the project. The Independent Advisory Board consisting of international research experts had a similar role. The research group consisted of researchers and students from AAU, SDC, and RCPH.

### 2.4. Intervention Development

**Figure 2:**
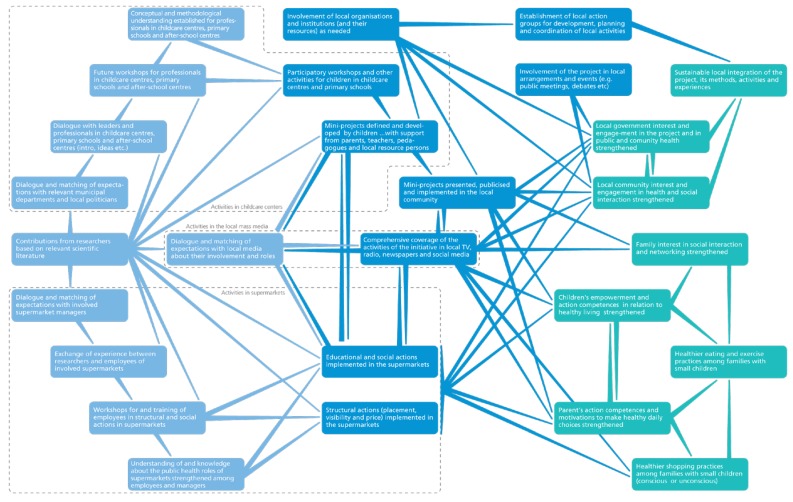
Programme theory for Project SoL.

The main focus in the development of the intervention was to promote healthier eating practices and increase physical activity (and decrease sedentary behaviour) by increasing the intake of fruit, vegetables, fish and whole grains; decreasing the intake of sugary beverages and sweets; and increasing physical activity through active play and the use of recreational areas. Furthermore, a main goal was to promote local ownership and sustainable integration of the project idea and health-promoting activities. With inspiration from Realist Evaluation [38] a program theory of the project was developed to guide both the development of interventions and the evaluation (see Figure 2). The program theory illustrates how intervention components were configured to address the core problems and causes and how the intervention components are believed to lead to proximal and distal outcomes.

**Figure 3:**
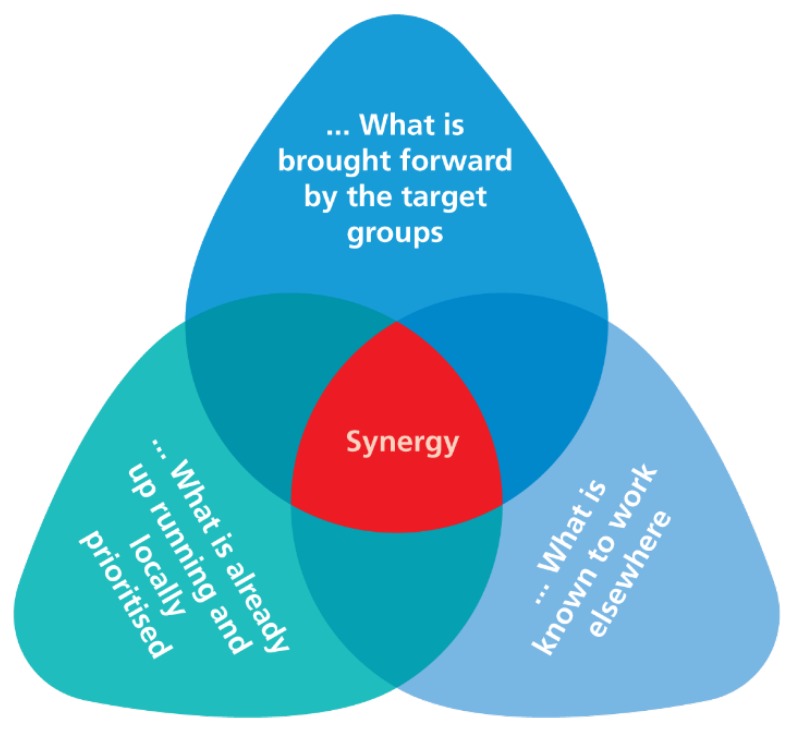
The development of activities was based on a balance between what was brought forward by the participating groups, what was known to work elsewhere, and what was already up running and prioritized locally. Activities that encompassed all three spheres provided maximal synergy and, thereby, impact.

By consistently using participatory approaches to engage with children, families, and professional stakeholders of the local community, their insights and aspirations for social action and health promotion were expected to influence and inform the development of specific intervention components. At the same time, it was prioritized that the intervention should be based on existing scientific evidence. Thus, as illustrated in Figure 3, the development of the activities was based on three sources of inspiration: (1) the perceived needs and ideas of target groups and professional stakeholders; (2) the wider priorities and development agendas of the local communities; and (3) the evidence-based knowledge and experience of the researchers. By consistently using all three sources of inspiration in interactive processes among project stakeholders and target groups, it was possible to create synergy and optimal opportunities to obtain effects and sustainability of the interventions. The researchers contributed with theory and evidence-based knowledge on what has worked (or did not work) in similar settings elsewhere. Moreover, the citizens and professional stakeholders contributed with knowledge and experience on what might be socially, culturally and financially acceptable, as well as knowledge about local development priorities, ongoing initiatives, resources, and willingness to explore local change potentials for strengthened health promotion action.

**Figure 4:**
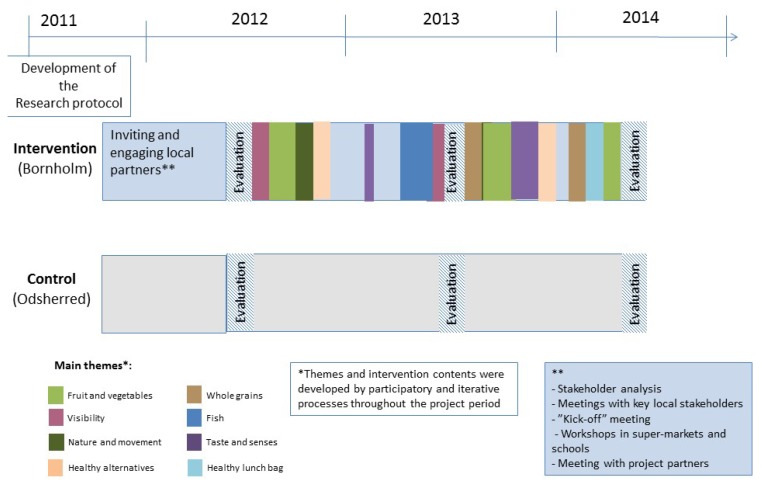
The timeline of Project SoL and the overall themes that were implemented.

We applied an action research approach to the overall development of the interventions [39,40]. Some intervention themes and components were proposed by researchers, whereas others were proposed by the local stakeholders and negotiated to fit into the overall intervention framework. In accordance with the action research methodology, the project implemented iterative cycles of participatory intervention development, assessment, and adjustment in which ideas were generated and knowledge was shared among researchers, local professional stakeholders, and citizens in the continued search for ways to improve health promotion in the local community [39,40]. The timeline and intervention themes of the Project SoL are illustrated in Figure 4.

Initially, a basis was established for local ownership of developing and implementing health promoting activities that were relevant and meaningful for the targeted families. This process began with initial meetings with municipality officials and politicians to obtain official local support for the project. Afterwards meetings were arranged with professionals from supermarkets, childcare centres, schools, and local media, in addition to relevant local NGOs (e.g., the local branches of The Danish Heart Foundation and the Danish Cancer Society). After a few months all relevant stakeholders were invited (through the local newspapers and personal invitations) to participate in a joint “kick-off” meeting with the purpose to establish and consolidate the local partnership and define common goals for the project. In the subsequent months several workshops and meetings were held with central employees from the selected supermarkets, childcare centres and schools as well as the local media to concretize the ideas and to plan for action.

### 2.5. Intervention Implementation

Generally, the activities aimed at promoting health by increasing knowledge, awareness, action competences, participation, integration, and social cohesion, as well as modifying the overall policies and structures to ensure that they supported healthier choices and, thereby, contributed to reducing social inequality. The intervention consisted of various types of activities and initiatives developed and implemented in collaboration with local stakeholders and citizens. Intervention components included “traditional” health promoting activities such as mass communication through mass and social media, brochures, recipes and posters, educational activities for children and their families and professionals in childcare centres, schools and supermarkets, and structural changes in schools, childcare centres and supermarkets. Additionally, mental and social well-being were promoted by activities, such as outdoor activities in nature, establishing aesthetic environments, tasting good food, exploring the senses, having fun, and building strong social networks.

To ensure coordination and integration of activities across settings and communities, common overall themes were selected and addressed based on the ideas and interests of the local partners and by the focus of the project. Hence, activities varied from setting to setting, and also from community to community, depending on the motivation and ideas of the stakeholders in different settings/communities, but were simultaneously implemented within the same overall theme both in supermarkets, childcare centres, and/or schools and mass media.

Overall themes included “taste and senses”, “active play”, “visibility”, “fruit and vegetables”, “whole grains”, “fish”, “nature and movement”, “nature as a pantry”, and “healthy alternatives”. The themes and activities were implemented in different ways in the three local communities on Bornholm as illustrated in Appendix A. To illustrate the process and intervention approach, the “Fish theme” is described in more detail in Box 1.

Box 1 **The Fish Theme—an example of a common theme across settings and communities.**

The fish-theme was chosen as a common theme in spring 2013. The theme was chosen because of the strong tradition of fishery as a profession on Bornholm, and the healthy benefits of eating fish, but also because the managers in the supermarkets repeatedly mentioned difficulties in selling fish in the supermarket combined with the fact that less fish can be bought directly from the harbour today because of the decline in fishing near Bornholm. The theme ran from April to June 2013 and the different activities were initiated both by local partners and by the project team.

In the media the fish-theme was covered in many different ways, including families cocking fish on the harbour during a big three-day event among fishermen, local fishermen were interviewed, and healthy fish recipes were posted on Facebook. Furthermore, the mass media (both television and local papers) covered the different activities happening in the local communities.

Children in childcare centres went for trips to the beach to select things from the ocean, and trips to the local fish smoking house; a local fisherman visited the childcare centres to tell about his work; fish was barbequed at parties in the childcare centres; fish were cooked in different ways and served for the children; children drew fish to decorate the local supermarkets. The children went to the supermarket with the fish they made and looked for fish to eat in the store.

In the supermarket fish products were placed more centrally in the store, the store was decorated with the children’s drawings, recipes with fish were placed together with the fresh or canned fish.

In the local community traditional recipes with fish and personal histories of the fishery were collected and posted on Facebook and in the mass media.

**Figure 5:**
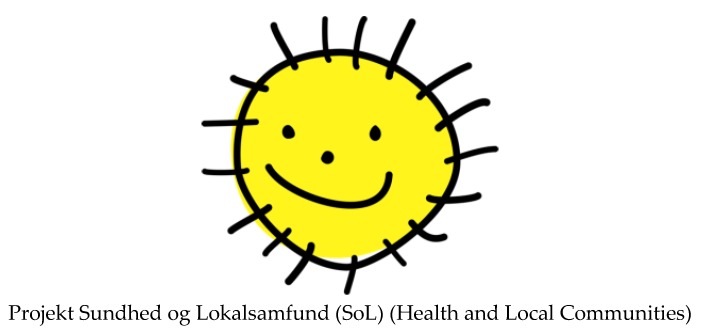
The SoL logo.

To promote local engagement, local visibility of the project was given a high priority. This included efforts to establish a positive image or a “brand” around the project. As described earlier “SoL” is an abbreviation of the Danish “**S**undhed **o**g **L**okalsamfund and “sol” is the Danish word for “sun”. Therefore, a local child’s drawing of a smiling sun was adapted graphically and used as the logo for the project on posters, letters, T-shirts, badges, key hangers, buses and shelf-talkers. The SoL logo is shown in Figure 5.

### 2.6. Promoting Sustainability

**Figure 6:**
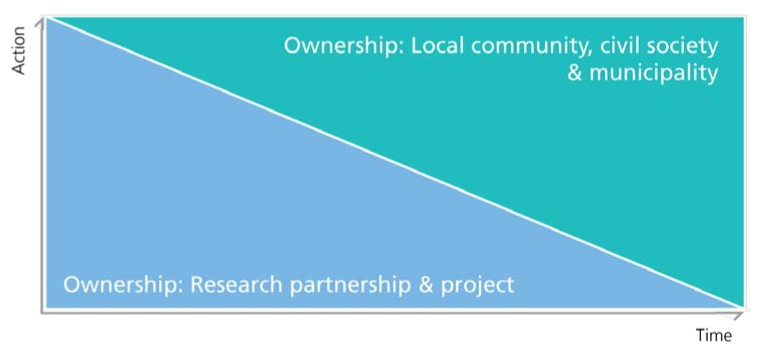
Illustration of the intended process towards greater local ownership of the project idea and health promoting activities.

Securing local ownership and long-term anchoring of the intervention was a key goal of Project SoL (Figure 6). To achieve this, a number of initiatives and steps were taken. In addition to the participatory approach, the employment of a local coordinator should ensure local engagement and relevance of the intervention. The local coordinator was a native citizen and, hence, she knew Bornholm and several of the local residents very well. This ensured local coordination on a daily basis but also provided the research group with important knowledge about local norms, traditions, and practices. Moreover, it minimized the distance between the project and the population on Bornholm by providing “a touch of local identity” in the communication and collaboration with local stakeholders. Sustainability was also promoted through training and capacity building of professionals and families at the local level. This was done through technical support, training, instruction, and inspiration from the research group and from external resource persons. Examples include:

- a health-educational staff course for employees in supermarkets;
- teaching principles of “nature fitness” to professionals from childcare centres, schools, and families;
- inspiration for healthy lunch boxes to families, children and professionals from childcare centres; and
- sensory education and cooking workshops for children, customers in supermarkets, and professionals in schools and child care centres.

Furthermore, the project sought to create a structure that would promote sustainability of the intervention beyond the lifespan of the project. Therefore, the formation of Local Action Groups (LAGs) in each of three selected local communities mid-term in the intervention period served as coordination and mobilization forums for community arrangements. The initiatives were based on local needs and priorities and planned and implemented through voluntary engagement. The broad representation of participants in the LAGs allowed for the implementation of activities that were community-based and community-involving rather than being confined to one setting. The establishment of the LAGs was, therefore, an important operational step, which fostered synergistic actions across settings and helped optimize their local relevance, integration and sustainability.

Finally, efforts were made to involve the public administration and local government of the municipality to create ownership around the project especially in the health department of the municipality as well as among local politicians. Towards the end of the project this resulted in the formalization of Project SoL’s approach and principles in the strategy for prevention and health promotion of the Regional Municipality of Bornholm. Thereby, sustainability of the project was further strengthened through wider political support and integration in the municipality. 

## 3. Discussion and Lessons Learned

This paper describes the processes related to the development and implementation of the intervention component of Project SoL. The paper is Part 1 of the two complementary papers described the intervention and research design of Project SoL. Thus, the paper by Mikkelsen et al. (Part 2) describes the equivalent research and evaluation framework and activities of the project. Project SoL builds on the supersetting approach [31], which is a conceptual framework for developing and implementing ecological, complex, and integrated activities across the boundaries of settings and stakeholders in local communities. The supersetting approach has been developed by the research partners of Project SoL and was implemented and tested for the first time by the project. The supersetting approach proved to be a very useful and relevant conceptual framework and strategic tool. It had the ability to facilitate the mobilization of local resources, to foster a high degree of local ownership, and to promote the development of locally-relevant activities of high intensity.

Project SoL differs from most other projects by including a local TV station as a formal project partner, and by developing and implementing coordinated and integrated activities in local mass media, social media, supermarkets, schools, after school clubs and childcare centres using a combination of different prevention strategies. Finally, Project SoL used a unique combination of a controlled intervention design for evaluating effects of the intervention and central principles from participatory methods for involving professional stakeholders and citizens at the community level.

### 3.1. Community-Based Multi-Component Interventions

Project SoL adds to the growing number of complex community-based health promoting interventions and projects that have been conducted over the past decades [41,42,43,44]. Overall, emerging evidence supports the implementation of integrated and coordinated interventions implemented in multiple community settings simultaneously, including a variety of intervention components, involving multiple stakeholders and implemented in numerous settings that are relevant to the everyday life context of citizens [23,26,27,28,29,30,42,43,44,45,46]. This kind of intervention program has greater intensity and, therefore, the potential to promote sustainable change. The feasibility and effectiveness of these interventions in promoting healthy eating and physical activity among children have been demonstrated in some earlier studies [47,48]. However, these studies mostly used primary schools as the dominant setting, and adiposity as the main outcome measure [48,49]. Two other examples that in many ways are similar to the Project SoL are Children’s Healthy Living for Remote Underserved Minority Populations of the Pacific [46] and B’More Healthy Communities for Kids: A multi-level obesity prevention program for low-income, urban African American children [23]. From Australia, the Romp and Chomp study [49] and the Be Active Eat Well study [50] are two other large and good examples of multi-component, multi-level community-based studies with the aim to reduce childhood obesity. Similar to Project SoL, these studies actively engaged a broad range of local stakeholders and communities in the development and implementation of intervention components while maintaining a strong research design. The projects described by Gittelsohn et al. [23] and Braun et al. [46] also implemented multiple activities across multiple levels and settings in a coordinated manner aiming at enhancing intervention intensity and effect, and they also used several different strategies to target children and their families’ knowledge, attitudes, empowerment, and behaviour through education, awareness-raising, and information provision, as well as by modifying the food and physical activity environments through structural changes. The Australian studies focused especially on local capacity building, awareness, and changing policies [49,50]. All programs were seeking to sustain their impact after the end of the intervention by working with many stakeholders through policy changes and coalition-building while, at the same time, carrying out their evaluation and other research tasks. However, overall, earlier studies are mainly implemented in American and Australian contexts, whereas European studies are scarce. Furthermore, Project SoL is the first study to use and test the supersetting approach as the overall theoretical framework of the intervention [31].

### 3.2. Collaboration with Multiple Stakeholders

Project SoL has shown that it is possible to establish a close collaboration between local stakeholders in the intervention clusters at schools, after-school clubs, childcare centres, supermarkets, and the local mass media, and that these stakeholders are willing to commit themselves to family-focused health promotion. However, to obtain this active participation and ownership among these very diverse stakeholders it was essential also to acknowledge the very different traditions and cultures that exist in their different settings. For instance, it became clear that the speed of decisions in the public administration of the municipality is very different from that in media and retail environments. To facilitate collaboration as a basis for developing coordinated and integrated intervention, the role of Project SoL’s researchers was not only to provide relevant theories and powerful evidence, but also to invest substantial amounts of time to develop and maintain good relations with key stakeholders. Moreover, the diverse contextual challenges and opportunities in the different settings needed to be taken into account.

An important role of the researchers was also to facilitate the sensation of a win-win situation among all stakeholders involved in the project. Thus, it was essential that the stakeholders could recognize the added value that could be obtained when stakeholders join in community-wide collaboration to the benefit of the entire community and its citizens. It soon became clear that scientific evidence about what works elsewhere was not always of interest to local project stakeholders in the processes of developing activities. However, in combination with locally-defined needs and priorities the literature describing experiences from other similar projects were found to be complementary and useful by the project. Interestingly, one of the factors that actually proved important during the process of engaging local stakeholders was the positive image that was created around the SoL logo. It became a way of showing that you, either as a citizen or a business partner, were part of a “club” that many people associated with something very positive and a local initiative with a local and recognizable identity that you would like to be associated with.

### 3.3. Collaboration with Local Authorities

Project SoL was carried out within the framework of the Regional Municipality of Bornholm and the schools and childcare centres involved in the project were administered by the municipality. Therefore, the project initially sought to gain legitimacy through dialogue and negotiation with the local authorities, including the mayor, various political councils in local government, and the administration of the municipality. This top-down approach was judged appropriate in spite of the overall participatory approach of Project SoL. This was done to ensure that the public institutions were allowed to engage in the project and because it was believed that ownership at the top level would strengthen the anchoring and sustainability of the project. Similarly, the collaboration with local supermarkets began with dialogue with the director at the chain level and the regional sales managers. As they decided to engage in the project, the local supermarkets were asked to participate by the regional sales managers.

The top-down approach resulted in some resistance, especially in the schools where teachers felt that their involvement was dictated from above, and researchers, therefore, had to invest a great deal of effort in the first phase of the project to promote local ownership, e.g. by providing resources and manpower to school activities. Retailers, on the other hand, were used to this kind of top-down decision-making and they actually had some resistance towards the participatory approach in Project SoL. Hence, some of the store managers expressed that they would prefer a strict plan for the intervention rather than dialogue and participation. Likewise, the mass media stakeholders found it difficult to commit to the participatory approach, but were eager to join in with ideas for activities and gain access to “news” and key persons of the project. 

### 3.4. Planning Based on Local Priorities, Ongoing Activities, and Existing Evidence

As described earlier, the development of the activities was based on three sources of inspiration: (1) what was brought forward by the target groups and professional stakeholders; (2) what was already up and running and locally prioritized; and (3) what was known to work elsewhere. The balance between these three sources of inspiration for developing activities differed throughout the project. In some cases, local ideas for activities were concrete and specific (e.g., in childcare centres). In other cases, stakeholders had no ideas and expected clear and concrete input and plans from the researchers (e.g., in supermarkets). However, as the project’s principles of local participation and ownership became more evident among stakeholders the balance between the three sources of inspiration shifted towards increasing engagement of local stakeholders in providing ideas and suggestions for new activities. With the establishment of LAGs, the communities’ wider development agendas and priorities became even more evident because more diverse community stakeholders were included. Hence, more attention was paid to creating community and social cohesion rather than focusing narrowly on healthy lifestyle in terms of healthy eating and physical activity. Thus, the formation of LAGs increased the local relevance of the intervention and was, at the same time, considered an important strategy to foster sustainability of the project based on the rationale that local action groups as informal organizational structures embedded in the local community would safeguard the wider health-related community interests. However, towards the end of the project period it was clear that the local action groups were not sustainable unless supervised and coordinated. Therefore, dialogue was initiated with formal and well-established organizations embedded in the local community, including local city councils, business councils, and citizen associations. At the same time, negotiations were held with the public administration at the municipality level about intensifying their role in providing technical and legal support to the involved communities. 

## 4. Conclusions

We conclude that coordinated and integrated health promotion activities that are implemented together with multiple stakeholders and across multiple settings in the local community are much more powerful than individual activities carried out in single settings. Project SoL demonstrated that the supersetting approach is a useful conceptual framework for developing and implementing such a complex multi-component health promotion intervention in the local community and helps create the ownership that is needed to ensure the sustainability of intervention.

## Figures and Tables

**Table 1:** Important characteristics of the adult citizens (>16 years) in Bornholm and Odsherred municipalities compared to the Capital Region.

Category	Characteristic	Unit	Bornholm	Odsherred	Capital Region
Population and area	Population	1000	41	32	1.754
	Area, Square km	Km^2^	588	355	2568
Health status	Overweight, BMI > 25	%	50	53	41
	Diabetes	%	6.5	5.7	4.5
	High blood pressure	%	16	23	22
Health behaviour	Citizens with very unhealthy dietary habits	%	14	16	10
	Citizens with <30 min/day MVPA	%	36	41	31
	Citizens with self-perceived poor health	%	18	21	15
Socio-Economic Position (SEP)	Unemployed	%	26	28	19
	No vocational education	%	19	18	8

Abbreviations: BMI, Body Mass Index; MVPA, Moderate and vigorous physical activity. Source: Glümer et al. [33] and Poulsen et al. [32].

## Supplementary Materials

Supplementary File 1

## Abbreviations

AAU	Aalborg University
SDC	Steno Diabetes Centre
RCPH	Research Centre for Prevention and Health
LAG	Local Action Group
SoL	Health and Local Community—from the Danish ‘Sundhed og Lokalsamfund’

## Appendix A

**2012**				
**Year/Month**	**Name of activity**	**Objectives**	**Settings**	**Intervention Components (Principles and *Themes*)**
January–March	Kick-off partnership meeting	Strengthen partnership and define common goals and plans	Nature museum Bornholm	Public meeting and workshop (participation, integration and *visibility*)
April–June	Visit and international symposium on ’food architecture’	Exchange knowledge with international researchers, Brian Wansink and Adam Brumberg from Cornell University and create inspiration for supermarkets	Copenhagen, Bornholm, supermarkets on Bornholm	Public meeting and workshop (participation, integration, empowerment and *visibility)*
	Folk meeting (political festival)	Create awareness and debate about health in local community	Allinge Folkemødet on Bornholm	Taste activities, quiz and debate with professionals (*visibility*)
	Supermarket workshop	Empower supermarket employees in healthy choices	Supermarkets	Workshops (participation and integration)
July–September	Three future scenario workshops	Empower children in health perspectives	Grade 2, Nexø school, Pedagogues and teachers at Bornholm	Future scenario workshop (participation and empowerment)
	Kick-off party with the local community	Create awareness about Project SoL. Gather and involve the project partners, families and professionals of SoL-Bornholm	The local community, Almindingen, Bornholm	Party with taste activities, play, games, sports, health theatre (participation, *visibility, taste and senses, active play*)
	Children’s drawings	Create awareness on healthy diet and create activities across settings (between childcare centres institutions and supermarkets)	Supermarkets, childcare centers, Bornholm	Children create drawings and hang them in the local supermarkets of Bornholm (Integration, *visibility, fruit and vegetables*)
October–December	Partnership meeting	Status on the project and input from the partnership	Conference centre, Nexø	Partnership meeting (participation)
	Child festival in Hasle	Increase awareness and skills to make healthy lunches	Hasle sports arena, childcare centres and school	Make your own lunch, make a creative sun carpet (Empowerment, *visibility, whole grain, fruit and vegetables*)
	Nature as pantry	Use the nature to empower children within healthy living	childcare centres	Forrest trips with professionals including nature guides and an actor. (Empowerment, *nature and movement*)
	Treasure hunt	Increase awareness and make it fun to eat healthy	Supermarkets, familiesNexø, Allinge, Hasle	Treasure hunts in supermarkets to find healthy ingredients for making a soup (Integration, Empowerment, *fruit and vegetables*)
	Lunch box seminar	Increase awareness and skills to make healthy lunches	Supermarkets, childcare centresNexø, Allinge, Hasle	Make your own lunch in supermarkets (integration, empowerment, fruit and vegetables and whole grains)
	Christmas calendar	Inspire families to make healthy meals, snacks and drinks during December	Supermarkets, Facebook	Healthy recipes based on local input shared in supermarkets and on Facebook (*visibility, healthy alternatives*)
**2013**				
**Year/month**	**Name of activity**	**Objectives**	**Settings**	**Intervention components (principles and *themes*)**
January–March	Meeting with school administration	Involve the schools and plan interventions and activities within the schools	Schools Nexø, Allinge, Hasle	Meeting (participation, integration)
	Meetings with TV2 Bornholm	Involve the media partners and plan the media intervention	Mass media	Meeting (participation, integration)
	Meeting with parents at Nexø school	Involve the families in SoL-activities	School, Nexø	Meeting (participation)
	Meeting with The Regional Municipality of Bornholm	Involve the municipalities	Municipality	Meeting (participation)
	Meeting with COOP	Involve the supermarkets and plan the supermarket intervention	Supermarkets	Meeting (participation, integration)
	Seminar “Sundhedsfremme and lokal sammenhængskraft”	Share and discuss the ideas of SoL with national researchers	AAU, Copenhagen	Seminar for professionals ‘health promotion and local community cohesion’ (knowledge)
	SoL-week—Healthy living, Nexø school	Increasing awareness on healthy diet and PA	School, supermarket, Nexø, mass media	Workshops and taste education in school tastings in supermarket, nature fitness (participation, integration, *taste and senses, nature and movement*)
	Lunch box circus	Increasing awareness on healthy diet and PA among families	Bornholms Højskole	Make your own “Healthy lunch box” day with play and presentations (knowledge, empowerment, *active play, fruit and vegetables, whole grains*)
April–June	Local group meetings	Involve the local communities in planning and execution of health promoting activities	Local community Nexø, Allinge, Hasle	Meetings (participation, integration)
	“Fish from a child perspective workshop”	Increasing awareness on healthy diet (Fish), planning TV programmes	Families, local community, Mass media	Healthy tips and fish recipes on Facebook, cooking workshop with children and families from Bornholm (empowerment, *Fish, visibility*)
	Healthy alternatives with Fish	Increasing awareness on eating fish. Motivate locals to share their knowledge about the local fish culture	Childcare centres, families, local community Nexø, Allinge, Hasle	Healthy tips & fish recipes on Facebook, visiting fish smoke house, cooking fish in childcare centres and with families (BBQ) in childcare centres (*Fish*)
	Healthy alternatives, Fish, Trolling	Increasing awareness on eating fish. Motivate locals to share their knowledge about the local fish culture	Childcare centres, Families, Local community, Allinge	Healthy tips and fish recipes on Facebook, cooking fish (BBQ) and orienteering at the harbour (empowerment, *Fish, visibility*)
	Healthy alternatives, Healthy snack	Increasing awareness on eating healthy snacks	Childcare centres, families, supermarkets, local community Nexø, Allinge, Hasle	Healthy snacks served in childcare centres and supermarkets (empowerment, integration, *Healthy alternatives*)
July–September	Healthy meal concepts	Promote healthy meal choices	Supermarket Nexø, Allinge, Hasle	Co-collation of food items for a healthy meal (integration, *Fish, Fruit and vegetables*)
	Candy-free checkouts	Collaboration with the supermarket staff to create healthy shops. Decrease the sale of candy and increase the sale of healthy snacks	Supermarkets Nexø, Allinge, Hasle	One candy-free checkout in four of the intervention supermarkets (participation, *healthy alternatives*)
	Focus on whole grains in the supermarkets (Harvest theme and inspiration for a healthy lunch)	Increasing awareness and motivation for eating whole-grain	Supermarkets, Mass media Nexø, Allinge, Hasle	Promotion of whole grains in supermarkets (*whole grains*)
	Harvest, Awareness week	Increasing awareness of SoL as well as awareness and motivation for healthy diet and PA	Childcare centres, schools, mass media Nexø, Allinge, Hasle	Taste activities in school and childcare centres (*whole grain, fruit and vegetables, visibility*)
	Harvest, “Carpenter festival”	Increasing awareness and motivation for healthy diet	Local community, school in Nexø	Cooking event with healthy foods arranged by the local action group in Nexø (integration, empowerment, *fruit and vegetables, whole grains*)
	Nature fitness	Increasing awareness and motivation for PA	Childcare centres, Nexø, Allinge, Hasle mass media	Activities with children and adults using the green areas around the childcare centres (empowerment, *Nature and movements*)
	Lunch bag activities	Inspiration and learning about how to make a healthy lunch box	Supermarkets, childcare centres, Mass media Nexø, Allinge, Hasle	Children making their own healthy lunch bag in the supermarket (empowerment, integration, *whole grains, fruit and vegetables*)
October–December	Nature fitness	Increasing awareness and motivation for PA	Childcare centres, Nexø, Allinge, Hasle Mass media	Activities with the children combined with a course on nature fitness for professionals (empowerment, integration, *nature and movements*)
	Harvest, Healthy, theme day Allinge	Increasing awareness and motivation for healthy diet	School, Allinge	Workshop in school, Orienteering with taste challenge (empowerment, *taste and senses, whole grains*)
	Harvest, Energy for life theme week with breakfast	Increasing awareness on healthy diet and PA	Schools, local community, Hasle mass media	Theme week in Hasle school (empowerment, *fruit and vegetables*)
	Harvest, Halloween party	Increasing awareness on healthy diet and PA	Childcare centres, local community, mass media Nexø	Halloween cooking event (soup) arranged by the local action group in Nexø (integration, *fruit and vegetables*)
	Child festival in Hasle	Increasing awareness on healthy diet and PA	Local community Hasle	Lunch workshop “make your own lunch” (integration, *whole grains, fruit and vegetables*)
	Lunch box seminars	Increasing awareness and skills to make healthy lunch	Childcare centres, supermarkets Nexø, Allinge, Hasle	Children making their own healthy lunch bag in the supermarket (empowerment, integration, *whole grains, fruit and vegetables*)
	Sensory, taste and cooking workshops	Increasing awareness on healthy diet	Childcare centres, schools Nexø, Allinge, Hasle	Taste and sensory education, cooking workshops in school and childcare centres (empowerment, *taste and senses*)
	Christmas calendar	Inspire families to make healthy meals, snacks and drinks during December	Supermarkets, Nexø, Allinge, Hasle Facebook	Healthy recipes based on local input shared on TV2s homepage and on Facebook (*visibility, healthy alternatives*)
	Christmas theme in Hasle school	Increasing awareness and motivation for healthy diet and PA	School, Hasle	Breakfast club, active week (empowerment, *whole grains, active play*)
**2014**				
**Year/Month**	**Name of activity**	**Objectives**	**Settings**	**Intervention Components (Principles and *Themes*)**
January–March	Kvickly’s yearly meeting	Inform the local community about SoL	Local community	Meeting in COOP and presentation of SoL (integration, *visibility*)
	Partnership meeting	Status on the project and input from partnership	Almindingen, Bornholm	Meeting with local partners (integration)
	Competition among supermarkets	Increasing sales of healthy foods in supermarkets. Engagement of supermarket employees	Supermarket, Nexø, Allinge, Hasle mass media	Competition on sales of fish, root fruits, and whole grain breakfast cereal (participation, *fish, whole grains, fruit and vegetables*)
	SoL by night	Increasing awareness on healthy choices in supermarkets	Supermarket, childcare centres, school Nexø, Allinge, Hasle Mass Media	Events in the supermarkets including activities for families about healthy lifestyle, tastings of local food products and a soup made by children from the local childcare centre, workshop for children with inspiration for healthy lunch bags (participation, empowerment, *fruit and vegetables, whole grains, fish*)
	Sensory-, taste and workshops	Increasing awareness on healthy diet	Schools and childcare centres Nexø, Allinge, Hasle	Taste and sensory education, cooking workshops in schools and childcare centres (empowerment, *taste and senses, fruit and vegetables*)
	Theme day healthy foods in Hasle	Increasing awareness and motivation for healthy diet	School, Hasle	Workshop in school, cooking healthy meals (empowerment, *fruit and vegetables, whole grains*)
	Theme on healthy breakfast	Increasing awareness and motivation for healthy diet	School, supermarket, mass media Nexø, Allinge, Hasle	
	Orienteering in the Dark, Allinge	Increasing awareness and motivation for healthy diet and PA	School, Local community Allinge	Orienteering race with taste samples and quiz arranged by the local group of Allinge (integration, *taste and senses, active play*)
	Folkemødet /Political festival	Create awareness and debate about health in local community	Allinge Folkemødet on Bornholm	Taste activities, quiz and debate with professionals (*visibility*)
	Lunch box workshop	Increasing awareness and skills to make healthy lunch	School Nexø, Allinge, Hasle	Lunch workshop “make your own lunch” (empowerment, *whole grains, fruit and vegetables*)
	Nexø city court (byting)	Local anchorage of intervention within the city court	Local community	Start-up of Nexø city court (integration)
	SoL finishing parties	Increase awareness of SoL and celebrate with the local community	Local community Nexø, Allinge, Hasle SoL partnership	Party in each of the three local communities (*visibility, fruit and vegetables, active play*)
April–June	Local group meetings	Involve the local communities in planning and execution of health promoting activities	Local community Hasle	Local group, Hasle (integration)
	Local group meetings	Strengthen the local anchorage of SoL	Local community Hasle, Nexø	Local group, Hasle and Nexø (integration)
	BRK	Strengthen the local anchorage of SoL	Local community	Local group, Hasle and Nexø (integration)

A public outdoor party with physical activity games, health theatre and a vegetable soup kitchen was held on 7 September 2012 to kick-start the intervention and to gather the approx. 400–600 children from childcare centres, schools and afterschool care as well as their teachers, pedagogies, supermarket employees, local sports clubs, parents and grandparents. The intervention components were designed to create synergy between the super-settings of the intervention (e.g., childcare, schools, afterschool care, supermarkets, and the local mass media), to promote healthy eating (e.g., vegetables, fruit, fish, and whole grains) and physical activity. Theme days and workshops focusing on health from a child perspective, taste education, healthy meals (e.g., breakfast, lunch, and social dinners), and physical activity (e.g., nature fitness) were arranged in childcare centres and schools and combined with additional events in the supermarket (e.g., tastings, ‘build your own lunch box’, soup treasure-hunts) and in the local community (e.g., lunch pack circus, night- orienteering race with taste samples). Final parties with all participants were held in each of the three local communities in April 2014 to hand over the project over to locals.
