# Supplementary material for: Project SoL—A Community-Based, Multi-Component Health Promotion Intervention to Improve Eating Habits and Physical Activity among Danish Families with Young Children. Part 1: Intervention Development and Implementation
Source: Int J Environ Res Public Health. 2018 May 28;15(6):1097. doi: 10.3390/ijerph15061097 (PMC6025396; doi:10.3390/ijerph15061097)
Supplement: Supplementary File 1 [file ijerph-15-01097-s001.pdf]

Appendix I. Description of the SoL interventions from 2012 to 2014 (text in italic marks represent the overall themes).

**2012**				
**Year/month**	**Name of activity**	**Objectives**	**Settings**	**Intervention components (principles and *themes*)**
January–March	Kick-off partnership meeting	Strengthen partnership and define common goals and plans	Nature museum Bornholm	Public meeting and workshop (participation, integration, and *visibility*)
April–June	Visit and international symposium on 'food architecture'	Exchange knowledge with international researchers, Brian Wansink and Adam Brumberg from Cornell University. And inspiration for supermarkets	Copenhagen, Bornholm, supermarkets on Bornholm	Public meeting and workshop (participation, integration, empowerment, and *visibility)*
	Folk meeting (political festival)	Create awareness and debate about health in local community	Allinge Folkemødet on Bornholm	Taste activities, quiz and debate with professionals (*visibility*)
	Supermarket workshop	Empower supermarket employees in healthy choices	Supermarkets	Workshops (participation and integration)
July–September	Three future scenario workshops	Empower children in health perspectives	Grade 2, Nexø school, Pedagogues and teachers at Bornholm	Future scenario workshop (participation and empowerment)
	Kick-off party with the local community	Create awareness about Project SoL; Gather and involve the project partners, families and professionals of SoL-Bornholm	The local community, Almindingen, Bornholm,	Party with taste activities, play, games, sports, health theatre (participation, *visibility, taste and senses, active play)*
	Children's drawings	Create awareness on healthy diet and create activities across settings (between childcare center institutions and supermarkets)	Supermarkets, childcare centers, Bornholm	Children create drawings and hang them in the local supermarkets of Bornholm (integration, *visibility, fruit and vegetables)*
October–December	Partnership meeting	Status on the project and input from the partnership	Conference centre, Nexø	Partnership meeting (participation)
	Child festival in Hasle	Increase awareness and skills to make healthy lunches	Hasle sports arena, childcare centres and school	Make your own lunch, Make a creative Sun carpet (Empowerment, *visibility, whole grain, fruit and vegetables)*
	Nature as pantry	Use the nature to empower children within healthy living	childcare centres	Forrest trips with professionals including nature guides and an actor (empowerment, *nature and movement)*
	Treasure hunt	Increase awareness and make it fun to eat healthy	Supermarkets, families	Treasure hunts in supermarkets to find healthy ingredients for making a soup (integration, empowerment, *fruit and vegetables)*
	Lunch box seminar	Increase awareness and skills to make healthy lunches	Supermarkets, childcare centres	Make your own lunch in supermarkets (integration, empowerment, fruit and vegetables, and whole grains)
	Christmas calendar	Inspire families to make healthy meals, snacks, and drinks during December	Supermarkets, Facebook	Healthy recipes based on local input shared in supermarkets and on Facebook (*visibility, healthy alternatives)*

**2013**				
**Year/month**	**Name of activity**	**Objectives**	**Settings**	**Intervention components (principles and *themes*)**
January–March				
	Meeting with school administration	Involve the schools and plan interventions and activities within the schools	Schools	Meeting (participation, integration)
	Meetings with TV2 Bornholm (2)	Involve the media partners and plan the media intervention	Mass media	Meeting (participation, integration)
	Meeting with parents at	Involve the families in SoL activities	School	Meeting (participation)

Appendix I. Description of the SoL interventions from 2012 to 2014 (text in italic marks represent the overall themes).

	Nexø school			
	Meeting with The Regional Municipality of Bornholm	Involve the municipalities	Local community	Meeting (participation)
	Meeting with COOP	Involve the supermarkets and plan the supermarket intervention	Supermarkets	Meeting (participation, integration)
	Seminar "Sundhedsfremme and local sammenhængskraft"	Share and discuss the ideas of SoL with national researchers	AAU, Copenhagen	Seminar for professionals 'health promotion and local community cohesion' (knowledge)
	SoL-week – Healthy living, Nexø school	Increasing awareness on healthy diet and PA	School, Supermarket, Mass media	Workshops & taste education in school<br>Tastings in supermarket, nature fitness (Participation, integration, *taste and senses, nature and movement)*
	Lunch box circus	Increasing awareness on healthy diet and PA among families	Bornholms Højskole	Make your own "Healthy lunch box" day with play and presentations (knowledge, empowerment, *active play, fruit and vegetables, whole grains)*
April–June	Local group meetings	Involve the local communities in planning and execution of health promoting activities	Local community	Meetings (participation, integration)
	"Fish from a child perspective workshop"	Increasing awareness on healthy diet (fish), planning TV programmes	Families, local community, Mass media	Healthy tips and fish recipes on Facebook, Cooking workshop with children and families from Bornholm (Empowerment, *Fish, visibility)*
	Healthy alternatives with Fish	Increasing awareness on eating fish. Motivate locals to share their knowledge about the local fish culture	Childcare centres, Families, Local community	Healthy tips and fish recipes on Facebook, visiting fish smoke house, Cooking fish in childcare centres and with families (BBQ) in childcare centres (*Fish)*
	Healthy alternatives, Fish, Trolling	Increasing awareness on eating fish. Motivate locals to share their knowledge about the local fish culture	Childcare centres, Families, Local community	Healthy tips and fish recipes on Facebook, Cooking fish (BBQ) and orienteering at the harbour (Empowerment, *fish, visibility)*
	Healthy alternatives, Healthy snack	Increasing awareness on eating healthy snacks.	Childcare centres, Families, Supermarkets, Local community	Healthy snacks served in childcare centres and supermarkets (empowerment, integration, *Healthy alternatives)*
July–September	Healthy meal concepts	Promote healthy meal choices	Supermarket	Co-collation of food items for a healthy meal (integration, *Fish, Fruit and vegetables)*
	Candy-free checkouts	Collaboration with the supermarket staff to create healthy shops. Decrease the sale of candy and increase the sale of healthy snacks.	Supermarkets	One candy-free checkout in four of the intervention supermarkets (participation, *healthy alternatives)*
July–September	Focus on whole grains in the supermarkets (Harvest theme and inspiration for a healthy lunch)	Increasing awareness and motivation for eating whole-grain	Supermarkets, Mass media	Promotion of whole grains in supermarkets (*whole grains)*
	Harvest, Awareness week	Increasing awareness of SoL as well as awareness and motivation for healthy diet and PA	Childcare centres, schools, Mass media	Taste activities in school and childcare centres (*whole grains, fruit and vegetables, visibility)*
	Harvest, "Carpenter festival"	Increasing awareness and motivation for healthy diet	Local community, school in Nexø	Cooking event with healthy foods arranged by the local action group in Nexø (integration, empowerment, *fruit and vegetables, whole grains)*
	Nature fitness	Increasing awareness and motivation for PA	Childcare centres, Mass media	Activities with children and adults using the green areas around the childcare centres (empowerment, *Nature and movements)*

Appendix I. Description of the SoL interventions from 2012 to 2014 (text in italic marks represent the overall themes).

	Lunch bag activities	Inspiration and learning about how to make a healthy lunch box	Supermarkets, mass media, childcare centres	Children making their own healthy lunch bag in the supermarket. (empowerment, integration, *whole grains, fruit and vegetables)*
October–December	Nature fitness	Increasing awareness and motivation for PA	Childcare centres, Mass media	Activities with the children combined with a course on nature fitness for professionals (Empowerment, integration, *nature and movements)*
	Harvest, Healthy, theme day Allinge	Increasing awareness and motivation for healthy diet	School	Workshop in school, orienteering with taste challenge (Empowerment, *taste and senses, whole grains)*
	Harvest, Energy for life theme week with breakfast	Increasing awareness on healthy diet and PA	Schools, local community, mass media	Theme week in Hasle school (empowerment, *fruit and vegetables)*
	Harvest, Halloween party	Increasing awareness on healthy diet and PA	Childcare centres, local community, mass media	Halloween cooking event (soup) arranged by the local action group in Nexø (integration, *fruit and vegetables)*
	Child festival in Hasle	Increasing awareness on healthy diet and PA	Local community	Lunch workshop "make your own lunch" (integration, *whole grains, fruit and vegetables)*
	Lunch box seminars	Increasing awareness and skills to make healthy lunch	Childcare centres, supermarkets	Children making their own healthy lunch bag in the supermarket (empowerment, integration, *whole grains, fruit and vegetables)*
	Sensory, taste. and cooking workshops	Increasing awareness on healthy diet	Childcare centres, schools	Taste and sensory education, cooking workshops in school and childcare centres (empowerment, *taste and senses)*
	Christmas calendar	Inspire families to make healthy meals, snacks and drinks during December	Supermarkets, Facebook	Healthy recipes based on local input shared on TV2s homepage and on Facebook (*visibility, healthy alternatives)*
	Christmas theme in Hasle school	Increasing awareness and motivation for healthy diet and PA	Schools	Breakfast club, active week (empowerment, *whole grains, active play)*
**2014**				
**Year/month**	**Name of activity**	**Objectives**	**Settings**	**Intervention components (principles and *themes*)**
January–March	Kvickly's yearly meeting	Inform the local community about SoL	Local community	Meeting in COOP and presentation of SoL (integration, *visibility)*
	Partnership meeting	Status on the project and input from partnership	Almindingen, Bornholm	Meeting with local partners (integration)
	Competition among supermarkets	Increasing sales of healthy foods in supermarkets. Engagement of supermarket employees	Supermarket, mass media	Competition on sales of fish, root fruits and wholegrain breakfast cereal (participation, *fish, whole grains, fruit and vegetables)*
	SoL by night	Increasing awareness on healthy choices in supermarkets	Supermarket, mass media, childcare centres, school	Events in the supermarkets, including activities for families about healthy lifestyle, tastings of local food products, and a soup made by children from the local childcare centre, workshop for children with inspiration for healthy lunch bags (participation, empowerment, *fruit and vegetables, whole grains, fish)*
	Sensory, taste. and	Increasing awareness on healthy diet	Schools and childcare	Taste and sensory education, cooking workshops in

Appendix I. Description of the SoL interventions from 2012 to 2014 (text in italic marks represent the overall themes).

	workshops		centres	schools and childcare centres (empowerment, *taste and senses, fruit and vegetables)*
	Theme day healthy foods in Hasle	Increasing awareness and motivation for healthy diet	School	Workshop in school , cooking healthy meals (empowerment, *fruit and vegetables, whole grains)*
	Theme on healthy breakfast	Increasing awareness and motivation for healthy diet	School, supermarket, mass media	
	Orienteering in the Dark, Allinge	Increasing awareness and motivation for healthy diet and PA	School, Local community	Orienteering race with taste samples and quiz arranged by the local group of Allinge (integration, *taste and senses, active play)*
	Folk meeting (political festival)	Create awareness and debate about health in local community	Allinge Folkemødet on Bornholm	Taste activities, quiz and debate with professionals (*Visibility)*
	Lunch box workshop	Increasing awareness and skills to make healthy lunch	School, Bornholm	Lunch workshop “make your own lunch” (empower-ment, *whole grains, fruit and vegetables)*
	Nexø city court (byting)	Local anchorage of intervention within the city court	Local community	Start-up of Nexø city court (Integration)
	SoL finishing parties (3)	Increase awareness of SoL and celebrate with the local community	Local community<br>SoL partnership	Party in each of the three local communities (*visibility, fruit and vegetables, active play)*
April–June	Local group meetings	Involve the local communities in planning and execution of health promoting activities	Local community	Local group, Hasle (integration)
	Local group meetings, (2)	Strengthen the local anchorage of SoL	Local community	Local group, Hasle and Nexø (integration)
	BRK	Strengthen the local anchorage of SoL	Local community	Local group, Hasle and Nexø (integration)

An official outdoor party with physical activity games, health theatre, and a vegetable soup kitchen was held on the 7 September 2012 to kick-start the intervention and to gather the approx. 400–600 children from childcare centres, schools, and afterschool care, as well as their teachers, pedagogies, supermarket employees, local sports clubs, parents, and grandparents. The intervention components were designed to create synergy between the super-settings of the intervention (e.g., childcare, schools, afterschool care, supermarkets, and the local mass media), to promote healthy eating (e.g., vegetables, fruit, fish, and whole grains) and physical activity. Theme days and workshops focusing on health from a child perspective, taste education, healthy meals (e.g., breakfast, lunch, and social dinners), and physical activity (e.g., nature fitness) were arranged in childcare centres and schools, and combined with additional events in the supermarket (e.g., tastings, ‘build your own lunch box’, soup treasure-hunts) and in the local community (e.g., lunch pack circus, night-orienteering race with taste samples). Final parties with all participants were held in each of the three local communities in April 2014 to hand the project over to the locals.
